# Expansive and Diverse Phenotypic Landscape of Field *Aedes aegypti* (Diptera: Culicidae) Larvae with Differential Susceptibility to Temephos: Beyond Metabolic Detoxification

**DOI:** 10.1093/jme/tjab179

**Published:** 2021-10-27

**Authors:** Jasmine Morgan, J Enrique Salcedo-Sora, Omar Triana-Chavez, Clare Strode

**Affiliations:** 1 Department of Biology, Edge Hill University, Ormskirk, UK; 2 Institute of Systems, Molecular and Integrative Biology, University of Liverpool, Liverpool, UK; 3 Instituto de Biología, Facultad de Ciencias Exactas y Naturales (FCEN), University of Antioquia, Medellín, Colombia

**Keywords:** insecticide resistance, *Aedes aegypti*, RNA-Seq, gene expression

## Abstract

Arboviruses including dengue, Zika, and chikungunya are amongst the most significant public health concerns worldwide. Arbovirus control relies on the use of insecticides to control the vector mosquito *Aedes aegypti* (Linnaeus), the success of which is threatened by widespread insecticide resistance. The work presented here profiled the gene expression of *Ae. aegypti* larvae from field populations of *Ae. aegypti* with differential susceptibility to temephos originating from two Colombian urban locations, Bello and Cúcuta, previously reported to have distinctive disease incidence, socioeconomics, and climate. We demonstrated that an exclusive field-to-lab (*Ae. aegypti* strain New Orleans) comparison generates an over estimation of differential gene expression (DGE) and that the inclusion of a geographically relevant field control yields a more discrete, and likely, more specific set of genes. The composition of the obtained DGE profiles is varied, with commonly reported resistance associated genes including detoxifying enzymes having only a small representation. We identify cuticle biosynthesis, ion exchange homeostasis, an extensive number of long noncoding RNAs, and chromatin modelling among the differentially expressed genes in field resistant *Ae. aegypti* larvae. It was also shown that temephos resistant larvae undertake further gene expression responses when temporarily exposed to temephos. The results from the sampling triangulation approach here contribute a discrete DGE profiling with reduced noise that permitted the observation of a greater gene diversity, increasing the number of potential targets for the control of insecticide resistant mosquitoes and widening our knowledge base on the complex phenotypic network of the *Ae. aegypti* response to insecticides.

Arboviral diseases including dengue, Zika, and chikungunya are amongst the most significant public health concerns worldwide. The geographical distribution and prevalence of these arboviruses have been increasing rapidly in recent years with the number of dengue infections reported to the World Health Organization (WHO) increasing 8-fold over the last 20 yr (WHO 2020). The most significantly affected world region is The Americas reporting 3,167,542 dengue cases, 181,477 chikungunya cases, and 35,914 Zika cases in 2019 alone (Villar et al. 2015, Guagliardo et al. 2019, Pan American Health Organization (PAHO) and World Health Organization (WHO) 2018, Bonilla-Aldana et al. 2020).

In the absence of effective vaccines for dengue, Zika, and chikungunya, disease control still relies on controlling mosquito vectors. Currently, this involves the use of insecticides including dichlorodiphenyltrichloroethane (DDT), pyrethroids, and organophosphates such as temephos, an approach that has not changed in over five decades of vector control programs (Maestre-Serrano et al. 2014). Temephos is one of the most used larvicides worldwide due to its ease of use, cost efficacy, and specificity towards the larval stages of mosquitoes (Fournier et al. 1992, Medicine 2021). Its pharmacological activities are related to the irreversible inhibition by phosphorylation of acetylcholinesterase (EC 3.1.1.7) (Fournier et al. 1992), a ubiquitous enzyme in Metazoan primarily expressed in the nerve endings and essential for termination of acetylcholine-mediated neurotransmission (Silman and Sussman 2005).

Temephos was first used as a method of controlling *Aedes aegypti* (Linnaeus) larvae in the early 1970s (Maestre-Serrano et al. 2014) with its continued use since leading to the development of resistance in *Ae. aegypti* in multiple regions of the world (Albrieu Llinás et al. 2010, Ocampo et al. 2011, Polson et al. 2011, Santacoloma et al. 2012, Grisales et al. 2013, Conde et al. 2015, Chediak et al. 2016, Goindin et al. 2017, Bharati and Saha 2018, Aponte et al. 2019, Saeung et al. 2020). The mechanisms conferring resistance to organophosphates have been well studied in other important vector mosquitoes but are less well understood in *Aedes* species despite their public health relevance. Mutations in the acetylcholinesterase (AChE) gene (*ace-1*) have been reported in temephos resistant insects (Fournier 2005) including the malaria vector *Anopheles gambiae* (Diptera: Culicidae) and the West Nile Virus vector *Culex pipiens* (Linnaeus, Diptera: Culicidae) (Weill et al. 2004b, Djogbénou et al. 2008, Tmimi et al. 2018). However, mutational alleles of the AChE gene are a rare finding in *Ae. aegypti* (Muthusamy and Shivakumar 2015, Hasmiwati et al. 2018) due to genetic constraints (Weill et al. 2004a).

A commonly reported insecticide resistance mechanism in mosquitoes is the increased expression of genes encoding for enzymes capable of metabolic detoxification of insecticides (Hemingway et al. 2004). Three main enzyme families have been associated with insecticide detoxification in mosquitoes: cytochrome P450 monooxygenases (P450s), glutathione S-transferases (GST), and carboxylesterases (CE). A total of 235 detoxification genes have previously been identified in *Ae. aegypti* (26 GSTs, 160 cytochrome P450s, and 49 CEs) (Strode et al. 2008). Overexpression of enzymes in all three of these groups has been associated with temephos resistance in *Ae. aegypti* (Marcombe et al. 2009, 2019; Grisales et al. 2013; Poupardin et al. 2014; Goindin et al. 2017). However, the genetic and phenotypic landscapes of insecticide resistance are wider and more complex. Insecticide resistance has also been associated with cuticular modification (through alteration of cuticular thickness or composition) (David et al. 2010, Riaz et al. 2013, Seixas et al. 2017, Balabanidou et al. 2018) and behavioral avoidance (Sukkanon et al. 2020). In those reports, comprehensive gene expression profiling has shown great discriminatory and quantitation power for identifying a wider range of potential genes involved in insecticide resistance responses (David et al. 2010, Riaz et al. 2013, Faucon et al. 2017, Seixas et al. 2017).

Next generation sequencing techniques, including RNA-Seq, provide a whole transcriptome approach to the identification of resistance genes with high sensitivity and specificity. RNA-Seq is now commonly used to investigate insecticide resistance in mosquitoes of medical relevance (e.g., *An. gambiae* (Bonizzoni et al. 2012), *Ae. albopictus* (Skuse, Diptera: Culicidae) (Xu et al. 2018)). In *Ae. aegypti* RNA-Seq has been utilized to characterize the gene expression changes associated with insecticide resistance developed through lab selection (David et al. 2014, Després et al. 2014, Cattel et al. 2021), however, this approach has sparsely been used for *Ae. aegypti* with field derived insecticide resistance (Faucon et al. 2017).

This study aimed to profile mechanisms of resistance to the larvicide temephos in natural populations of *Ae. aegypti.* The field samples originated from areas of differential arbovirus burden and incidence in Colombia. In a previous study, we stratified three regions in this country with distinctive arboviral disease incidence, climatic variables, and socio-economic profiles through a recent time window of 11 yr (Morgan et al. 2021). In the present work, *Ae. aegypti* mosquito samples from two of those regions, Bello and Cúcuta, which had the lowest and highest strata of disease burden, respectively were analyzed. The differential gene expression associated with the resistance to temephos was profiled in these two field populations of *Ae. aegypti* (field-to-field comparison) with a data triangulation against the gene expression of the lab-adapted *Ae. aegypti* reference strain New Orleans (NO).

The comparison of a field resistant population against a susceptible field population and a susceptible lab population allowed us to select for gene expression specifically related to the trait under study (resistance to temephos in circulating natural populations of *Ae. aegypti*) while minimizing the background noise from genotypic distance and phenotypic drifting of field mosquito populations. The present work illustrates two angles of observations in mosquito biology under selective pressure to the larvicide temephos: firstly, the potential mechanisms of insecticide resistance itself and secondly, the additional changes that field insecticide resistant larvae undergo when in transient exposure to the insecticide. The data presented significantly expands the hitherto known composition of the gene expression responses of *Ae aegypti* mosquitoes resistant to the larvicide temephos, with a granularity at the transcriptomic level that goes beyond detoxification genes.

## Materials and Methods

### *Ae. aegypti* Field Collection and Colonization

**Fig. 1.**
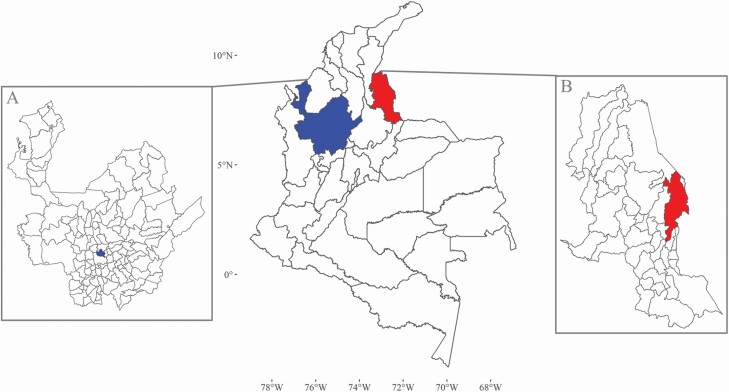
**The location of the two study sites.** Mosquito collections took place in these two locations, Bello and Cúcuta, within Colombia. Departments are the largest units of local government. (A) Department of Antioquia governs Bello. (B) Department of Norte de Santander has as its capital Cúcuta, a city to the East of this department on the border with Venezuela. Map base layers were obtained from https://data.humdata.org/dataset/colombia-administrative-boundaries-levels-0-3 covered by a Creative Commons Attribution 4.0 International (CC BY) License (https://creativecommons.org/licenses/by/4.0/legalcode). Map base layers were modified by the addition of colors.

*Ae. aegypti* were collected from the Colombian municipalities of Bello and Cúcuta (Fig. 1). These municipalities were previously shown to be distinct in the burden of *Ae. aegypti* borne disease, socioeconomic status, and climate (Morgan et al. 2021). Mosquito collections took place in 2016 (Bello) and 2017 (Cúcuta) with the assistance of personnel from biology and control of infectious diseases research groups (University of Antioquia) and vector-borne disease program staff within each municipality. Immature *Ae. aegypti* were collected from deployed oviposition traps (ovitraps) and reared to adults under standard conditions at Universidad de Antioquia, Colombia. Standard rearing conditions were 28 ± 1°C, 80 ± 5% relative humidity and a 12 hr light: 12 hr dark photoperiod. Reared adults were offered a bloodmeal and the eggs were collected for the establishment of colonies. Upon establishment of colonies, eggs were collected and sent to Edge Hill University, UK for insecticide resistance profiling.

### Larval Bioassays

Larvae for use in insecticide bioassays were reared under standard conditions within Edge Hill University Vector Research Group insectaries. Standard conditions were 27°C and 70% RH with an 11-hr day/night cycle with 60-min dawn/dusk simulation periods, using a lighting system of 4× Osram Dulux 26W 840 lights. Eggs were submerged in a hatching broth of 350 ml distilled H_2_O (dH_2_O), 0.125 g nutrient broth (Sigma–Aldrich, Darmstadt, Germany), and 0.025 g brewer’s yeast (Holland & Barrett, Ormskirk, UK) for 48 hr (Zheng et al. 2015). Larvae were fed ground fish food (AQUARIAN advanced nutrition) and raised until third to fourth instar. Larval bioassays were conducted following WHO standard test procedures (WHO 2016). Preliminary testing was conducted to identify the activity range of temephos to larvae from each of the study municipalities and a susceptible laboratory strain (New Orleans). The activity ranges, yielding mortality of 10–95%, for each *Ae. aegypti* population as identified by preliminary testing are displayed in Table 1. At least four replicates, each consisting of 20 third to fourth instar larvae, were conducted for each temephos concentration. Fresh insecticide solutions were made for each replicate using temephos (93.7%; Pestanal, Sigma–Aldrich Darmstadt, Germany) and acetone (□≥99.9%; Sigma–Aldrich, Darmstadt, Germany). Bioassays were conducted inside the insectaries under standard conditions and mortality was recorded after a 24-hr exposure period. Following WHO guidelines, moribund larvae were counted as dead. Controls were exposed to the acetone solvent only.

**Table 1.** **Temephos activity range yielding between 10-95% mortality to each mosquito population.** Results of preliminary testing to identify the activity range of temephos to each mosquito population. Activity ranges displayed in parts per million (ppm)

Population	Temephos activity range (ppm)
Bello	0.008–0.05
Cúcuta	0.02–0.10
New Orleans	0.004–0.008

### RNA Extraction and cDNA Synthesis

#### *Aedes aegypti* Sample Groups

**Fig. 2.**
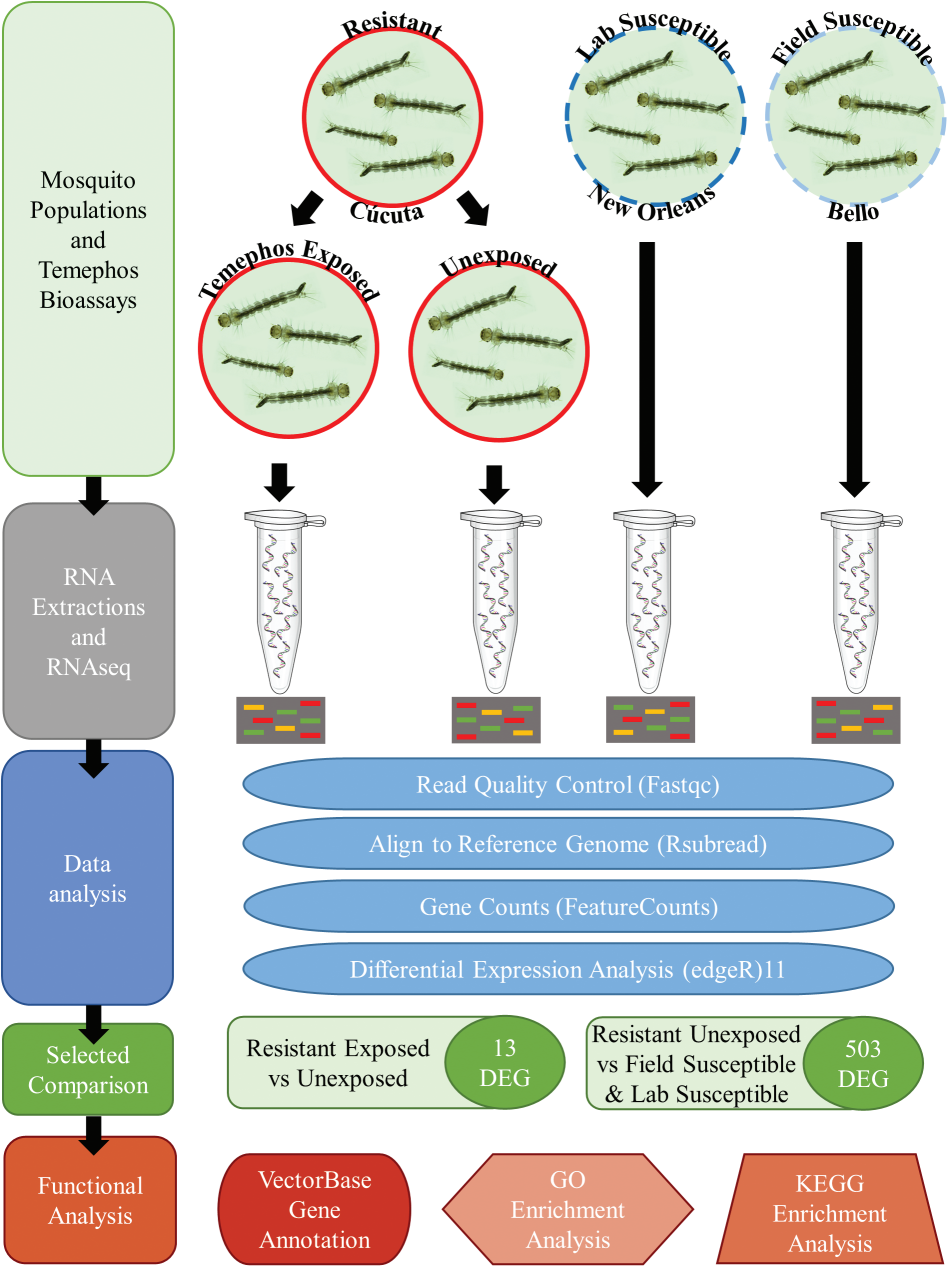
**Block diagram of our experimental approach**. This study concerned the larval stages of the mosquito *Ae. aegypti*. Field samples denoted as Resistant originated from the Cúcuta, Colombia population. The Susceptible samples had dual origin: Field samples from Bello, Colombia (Field Susceptible) and the New Orleans reference lab strain (Lab Susceptible). The total RNA sequenced and mapped (Data analysis) originated from four different experiments (biological replicates) from each population. The gene expression levels of the Resistant samples compared against the Lab Susceptible and Field susceptible had at least 503 differentially expressed genes (DEG). The Resistant samples of larvae transiently exposed to temephos had 13 DEGs in comparison to the unexposed larvae. The functional annotation for the DEG sets was carried with several different repositories: VectorBase, Gene Ontology (GO), and KEGG Enrichment Analysis.

Following the larval bioassays, field samples were categorized as resistant (Cúcuta) or susceptible (Bello). In the latter category were also samples from the lab adapted reference strain New Orleans. The Cúcuta temephos resistant samples were further divided into two groups: one exposed to temephos (for 24 hr) immediately before sampling for RNA extraction, and a control group of no temephos exposure (unexposed) (Fig. 2). For each group (field susceptible (FS), lab susceptible (LS), field resistant exposed (FRE) and field resistant unexposed (FRU) RNA extractions were carried out from four different larvae batches considered here as biological replicates.

#### Standard Rearing of *Aedes aegypti* for RNA Extraction

*Ae. aegypti* were reared to fourth instar larvae following a standard rearing protocol and under standard conditions within Edge Hill University Vector Research Group insectaries. Standard conditions were 27°C and 70% RH with an 11-hr day/night cycle with 60-min dawn/dusk simulation periods, using a lighting system of 4× Osram Dulux 26W 840 lights. Eggs were submerged in a hatching broth of 350 ml dH_2_O, 0.125 g nutrient broth (Sigma–Aldrich, Dorset, UK), and 0.025 g brewer’s yeast (Holland & Barrett, Ormskirk, UK) for 48 hr (Zheng et al. 2015). Once hatched, larvae were reared at a density of one larva/ml in dH_2_O and fed ground fish food (AQUARIAN advanced nutrition) at increasing quantities per day (day 3 = 0.08 mg/larva, day 4 = 0.16 mg/larva, day 5 = 0.31 mg/larva) (Carvalho et al. 2014). Six days after egg submersion larvae were subjected to an insecticide bioassay in batches of 25 larvae in 100 ml dH_2_O, for 24 hr (WHO 2016).

#### Temephos Exposure Assays for Resistant Larvae

Larvae in the resistant exposed group were exposed to temephos at the LC_50_ of 0.06 ppm (Fig. 2). Larvae in all unexposed groups were exposed to the equivalent volume of acetone (the solvent used in temephos solutions). After the 24 hr bioassay larvae were taken for RNA extraction. For each experimental group there were four independent replicates, conducted using eggs from different batches and rearing and extraction conducted on different days. Egg submersion, feeding, bioassays, and RNA extraction on all replicates were all conducted at the same times of day.

#### RNA Extraction

Larvae were homogenized using QIAshredders (Qiagen, Manchester, UK) then RNA extracted using PicoPure RNA Isolation Kit (Arcturus Bioscience, Mountain View, USA) following the manufacturers’ protocols. RNA was extracted from a total of 20 individual larvae per biological replicate, with four larvae per column and the total RNA was then pooled. RNA quality and quantity were assessed using an Agilent 2100 bioanalyzer. The temephos exposed population had 12–16 larvae per replicate due to mortality during bioassays.

#### Library Preparation and Sequencing

Library preparation and sequencing was conducted at Polo d’Innovazione di Genomica, Genetica e Biologia, Italy. Libraries were prepared following the QIAseq Stranded mRNA Select Kit Handbook (June 2019) for Illumina Paired-End Indexed Sequencing (Qiagen 2019). Libraries were validated using the Fragment Analyzer High Sensitivity Small Fragment method to assess size distribution and quantified using a Qubit 3.0 Fluorometer. Indexed DNA libraries were all normalized to 4 nM, pooled in equal volumes, and then loaded at a concentration of 360 pM onto an Illumina Novaseq 6000 S1 flowcell, with 1% of Phix control. The samples were sequenced using the Novaseq 6000 standard workflow with 2 × 150 bp pair end run. The experimental design for this study is outlined in Fig. 2. The raw reads obtained through RNA-Seq are deposited in NCBI’s sequenced read archive (Accession PRJNA730411).

### Data Analysis

#### Bioassay Data Analysis

Larval 50% and 95% lethal concentrations (LC_50_ and LC_95_) and their 95% confidence limits (*P* < 0.05) were calculated using probit analysis according to Finney (1947) (Ross and Finney 1972) using the LC_probit function in the R ecotox package (version 1.4.0) (Hlina et al. 2019). Abbot’s correction (Abbott 1987) was not applied due to the control mortality never exceeding 10%. Resistance ratios (RR_50_ and RR_90_) were calculated to assess temephos resistance by comparison of LC_50_ and LC_90_ for *Ae. aegypti* from each field location to those of the susceptible laboratory strain (New Orleans). Resistance ratios were defined as susceptible (<5-fold), moderate resistance (5–10-fold), and high resistance (>10-fold) following WHO guidelines (WHO 2016). Statistical analyses of bioassay data were conducted using R statistical software (version 3.6.1) (R Core Team 2020).

#### RNA-Seq Data Quality Control, Mapping, and Differential Gene Expression

Analyses of RNA-Seq data were conducted using the Linux command line (Ubuntu 18.04) and R statistical software (version 4.0.3) (R Core Team 2020). Sequence read quality was assessed using FastQC (version 0.11.3). Reads with quality scores less than 30 and lengths less than 50 bp were trimmed using cutadapt (version 2.10) with Python (version 3.8.3). Read quality was then reassessed using FastQC to ensure only high-quality reads remained. Cleaned reads were mapped to the *Ae. aegypti* LVP_AGWG reference genome (version AaegL5, GenBank: NIGP00000000.1) using Rsubread (version 2.2.6) (Liao et al. 2019). The resultant BAM files were sorted and indexed using samtools (version 1.11). Alignment quality metrics from Rsubread were visualized using R’s plotting function. Gene count tables were generated using Rsubreads. Read counts were normalized using the trimmed mean of M values method (Robinson and Oshlack 2010) in edgeR (Version 3.30.3) which accounts for library size and expression bias in RNA-Seq datasets (Robinson et al. 2010). TMM normalization was conducted between groups in each comparison rather than globally across all samples to ensure within-group variability could be detected. Differential gene expression was then calculated using a Quasi-likelihood negative binomial generalized log linear model (edgeR). Quasi-likelihood error family was selected due to its ability to account for uncertainty in dispersion. Counts per million (CPM) were calculated in edgeR and reads per kilobase million (RPKM) were calculated in edgeR using transcript lengths obtained from Enseml Metazoa (LVP AGWG (aalvpagwg_eg_gene) dataset) using biomaRt (version 2.44.4). Transcripts with fold-change >2 and FDR <0.05 were selected for gene ontology and KEGG pathway enrichment analyses, these thresholds were used to select genes with significant and larger differential expression.

#### Gene Ontology and KEGG Pathway Enrichment Analyses

Gene ontology (GO) category assignments were obtained from Ensembl Metazoa using biomaRt (version 2.44.4) (Durinck et al. 2009) and KEGG pathway assignments from Kyoto Encyclopedia of Genes and Genomes using KEGGREST (1.28.0) (Tenenbaum 2020). GO and KEGG enrichment analyses were conducted using GOseq (version 1.40.0), which allows for the correction of biases arising from the variable transcript lengths in RNA-Seq data (Young et al. 2010). Enrichment scores were calculated using the Wallenius method within GOseq. *P*-values were then corrected for multiple testing using the Benjamini–Hochberg method in the p.adjust function (R Core Team 2020). GO categories and KEGG pathways with corrected *P* values <0.05 were considered significantly enriched. Enrichment percentage was calculated as a ratio of the number of differentially expressed genes within each category to the total number of genes within that category.

## Results

### Temephos Susceptibility of *Ae. aegypti* Field Isolates and the Reference Strain

The lethal concentrations (LC_50_) of temephos were 0.019 *ppm* (95% Cl 0.016–0.029) and 0.060 *ppm* (Cl 95% 0.052–0.070) for Bello and Cúcuta, respectively. This corresponded to resistance ratios of 2.6 and 8.0 when compared to the New Orleans susceptible laboratory strain (LC_50_ = 0.008; Cl 95% 0.006–0.012). The LC_95_ for Bello was 0.055 (Cl 95% 0.034–0.292), 2.8-fold higher than New Orleans (LC_95_ = 0.020; Cl 95% 0.012–0.21). Cúcuta had an LC_95_ of 0.182, 9-fold higher than New Orleans (Table 2). Following the WHO guidelines (WHO 2016) larvae from Bello were considered susceptible to temephos and denoted as the field susceptible (FS) population whist the larvae from Cúcuta were moderately resistant to this larvicide and denoted as the field resistant (FR) population (Table 2). New Orleans is referred to as the lab susceptible (LS) population.

**Table 2.** **LC**_**50**_ **and LC**_**95**_ **of Bello, Cúcuta, and New Orleans *Ae. aegypti* larvae to temephos.** Temephos bioassays showing LC50, LC95, and their 95% confidence limits, calculated using probit analysis. Resistance ratios (RR) were calculated as a ratio of the lethal concentration (LC50 and LC95) of each population compared to the lab susceptible (New Orleans) strain. Bello and New Orleans are both susceptible to temephos whilst larvae from Cúcuta are resistant. SE = standard error

Population	*n* ^*a*^	LC50 (95% CI)	LC95 (95% CI)	Slope ± SE	RR50	RR95
Bello (Field susceptible)	580	0.0193 (0.0156–0.0293)	0.0554 (0.0340–0.2921)	3. 5929 ± 0.3503	2.6	2.8
Cúcuta (Field resistant)	400	0.0599 (0.0516–0.0698)	0.1818 (0.1357–0.3081)	3. 4115 ± 0.3465	8.0	9.0
New Orleans (Lab susceptible)	400	0.0075 (0.0064–0.0123)	0.0201 (0.0123–0.2075)		1.0	1.0

^*a*^ *n* = the total number of larvae tested across all replicates.

### RNA-Seq Mapping Summary

Three different populations of *Ae. aegypti* larvae were profiled using RNA-Seq: temephos field resistant (FR), field susceptible (FS), and the reference lab susceptible strain New Orleans (LS). The FR population was also split into two further groups as either exposed or not (unexposed) to temephos to determine further expression changes associated with insecticide exposure in already resistant populations (Fig. 2). Mosquito larvae from each of these four populations were grown in four different batches, one to four weeks apart, and were considered here four biological replicas. This generated a total of 16 sequenced samples.

Extracted total RNA was used to generate the Illumina RNA-Seq libraries that produced 65.7–120 million reads per sample with quality scores >30 and lengths >50 bp and 84.4% and 85.6% of those reads in each sample were successfully aligned to the reference genome (Table 3 and Materials and Methods). Using the most current gene model available for *Ae. aegypti,* LVP_AGWG reference genome which contains 19,381 open reading frames (version AaegL5, GenBank: NIGP00000000.1) the number of genes with successfully aligned reads ranged from 15,704 and 16,607 genes across all samples, corresponding to 81–86% of the total genes in the reference genome.

**Table 3.** **RNA-Seq sequencing data summary.** The total number of obtained reads after quality control and the percentage of reads that mapped to the *Aedes aegypti* reference genome. Quality control removed reads with quality <30 and lengths <50 bp

Group	Sample	Total reads (million)	Mapped to genome (%)
Field susceptible	Bello 1	93.6	85.70
	Bello 2	107.9	85.50
	Bello 3	104.0	86.21
	Bello 4	87.7	85.37
	**Bello Mean**	**98.3**	**85.70**
Field resistant	Cúcuta Exposed 1	95.3	84.42
	Cúcuta Exposed 2	120.0	84.67
	Cúcuta Exposed 3	65.7	84.77
	Cúcuta Exposed 4	84.5	85.93
	**Cúcuta Exposed Mean**	**91.4**	**84.95**
	Cúcuta Unexposed 1	78.6	84.35
	Cúcuta Unexposed 2	98.4	85.45
	Cúcuta Unexposed 3	111.2	84.90
	Cúcuta Unexposed 4	93.4	85.14
	**Cúcuta Unexposed Mean**	**95.4**	**84.96**
Lab susceptible	New Orleans 1	105.3	85.91
	New Orleans 2	94.7	86.54
	New Orleans 3	93.3	86.59
	New Orleans 4	87.0	86.54
	**New Orleans Mean**	**95.1**	**86.40**

### Overview of Differential Gene Expression

**Fig. 3.**
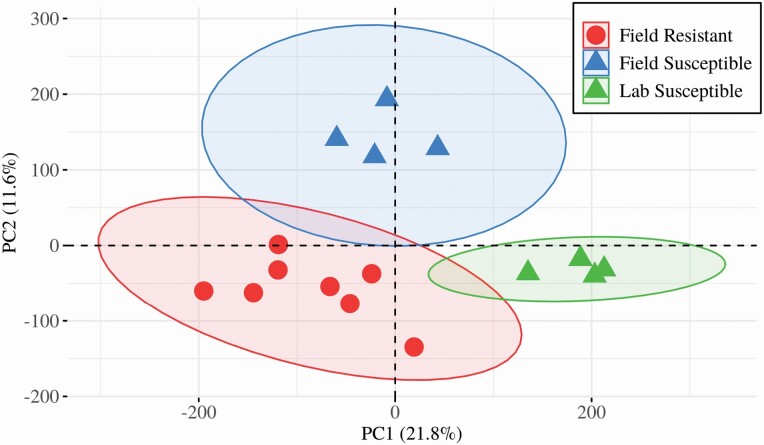
**Distribution of the transcriptomic profiles by sample groups.** The multidimensionality of the RPKM values calculated for each mapped transcript per sample was reduced by principal components analysis (PCA). The field samples were seen linearly distant from each other across one component whist the reference lab strain NO cluster (Lab Susceptible) separated from both field samples. The orthogonal dispersion of these samples allowed for the triangulation of the data as described in the main text.

**Fig. 4.**
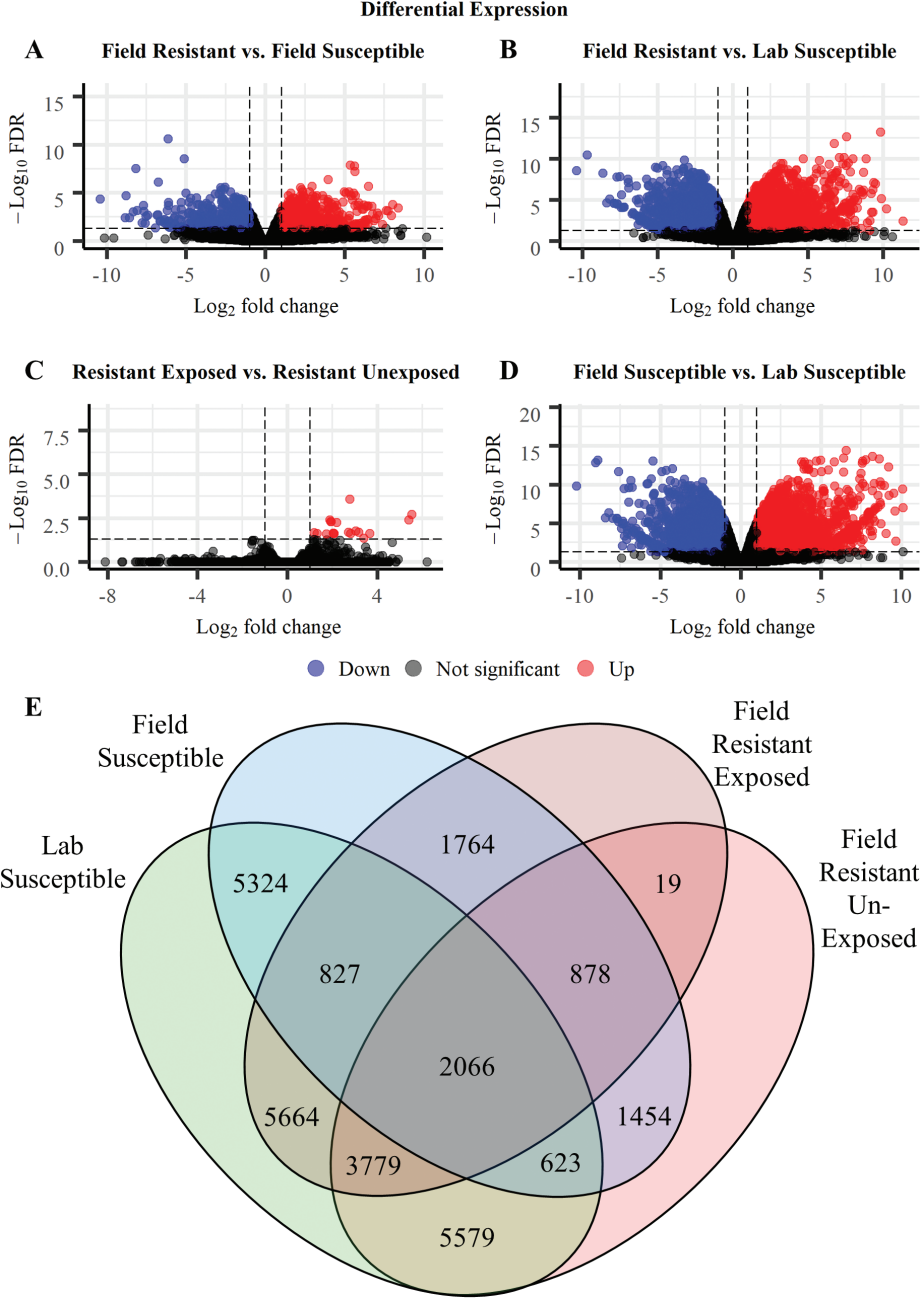
**Differential gene expression in the samples from field susceptible, lab susceptible, and field resistant populations.** (A–D) Differential expression of all transcripts including those over expressed, under expressed, and with no significant differential expression in the field resistant unexposed population compared to the field susceptible population (A) and susceptible lab population (B), the resistant temephos exposed population when compared to the resistant unexposed population (C) and in the field susceptible population when compared to the lab susceptible population (D). (E) The number of transcripts significantly differentially expressed (FC > 2, FDR < 0.05) between each of the experimental groups. The number of differentially expressed transcripts shown here (E) includes both significantly over and under expressed transcripts. The comparison groups and sample notation as detailed in Fig. 2.

Both field samples, susceptible and resistant (FS, FR) were equally and evidently distant, by RPKM number, to the samples of the lab strain New Orleans (LS) (Fig. 3). Crucially the temephos susceptible samples did not cluster together nor did the two field samples (Fig. 3). Field and lab strain samples were similarly distant in terms of differentially expressed (DE) transcripts: FS versus LS = 5324 DE transcripts (Fig. 4D and E), FR versus LS = 5579 (Fig. 4B and E). However, when comparing field samples (resistant and susceptible) the number of DE transcripts visibly lowered by four-fold to 1,454 (Fig. 4A and E). Therefore, the common practice in gene expression studies of comparing mosquito field samples to lab strains (e.g., Marcombe et al. 2009, Grisales et al. 2013, Dusfour et al. 2015, Ishak et al. 2017), would have generated an approximately 4-fold overestimation in the number of DE transcripts detected in the field resistant samples.

We also sought to investigate the gene expression changes in insecticide resistant larvae under transient exposure to insecticide. There were only 19 transcripts significantly differentially expressed in larvae within the resistant population which were exposed to temephos when compared to unexposed larvae from the same population (Fig. 4E). All 19 of those transcripts were overexpressed in the exposed group with no significant down regulated gene expression detected (Fig. 4C).

We addressed the issue of potential misrepresentation of gene expression metrics by triangulating both, the differentially expressed gene (DEG) sets and the RPKM counts between the field resistant (FR) samples against the field susceptible (FS) as well as the lab susceptible (LS) samples. The DEG set obtained contained transcripts which were found to be significantly differentially expressed with a fold change of >2 and a false-discovery rate (adjusted *P* value) of <0.05 in both comparisons. Under this threshold, a total of 623 (down from 1,454 transcripts in only the field-to-field comparison) transcripts covering 503 genes, were found differentially expressed in the field resistant population when compared to both the field and lab susceptible populations (Fig. 4E). This set of 503 genes comprised 301 overexpressed genes and 202 under expressed genes (Supp Table 1 [online only]). Of the 301 significantly overexpressed genes 239 were found in the category of protein coding genes: 88 annotated and 151 hypothetical genes. In the significantly under expressed gene set 166 were protein coding with 75 annotated and 91 hypothetical genes (Supp Table 1 [online only]). The significant differentially expressed genes also included 55 overexpressed genes encoding for long noncoding RNA (lncRNA) and 30 under expressed lncRNA genes in the temephos resistant larvae (Supp Table 1 [online only]).

### Gene Ontology and KEGG Pathway Enrichment

**Fig. 5.**
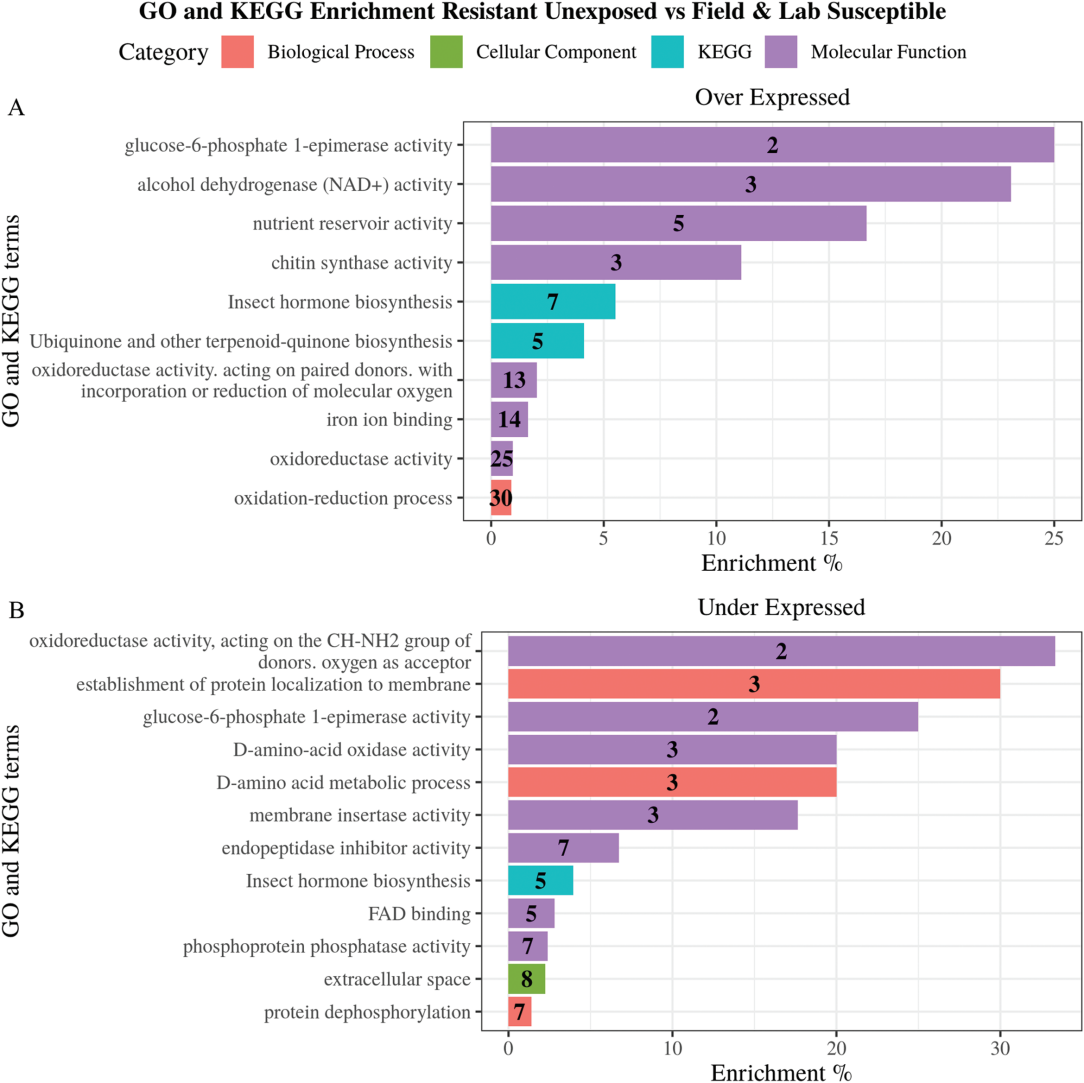
**GO terms and KEGG pathways enriched in the resistant population when compared to both field and lab susceptible populations.** GO terms and KEGG pathways found to be significantly enriched (*P* < 0.05) following Benjamini Hochberg correction in the significantly over (A) and under (B) expressed transcripts. Enrichment percentage was calculated as the number of differentially expressed transcripts in each category/pathway divided by the total number of transcripts in the same category/pathway. Number in bars indicates the number of differentially expressed transcripts in each category.

All 623 transcripts with fold-change >2 and FDR <0.05 were selected for gene ontology (GO) and KEGG pathway enrichment analyses. GO categories and KEGG pathways with corrected p values (FDR) <0.05 were considered significantly enriched. Gene ontology and KEGG enrichment analyses conducted on the 301 significantly over expressed genes identified eight significantly enriched GO categories; one involved with biological processes, oxidation–reduction processes (GO:0055114), and seven associated with molecular functions (GO:0045735, GO:0016705, GO:0004100, GO:0004022, GO:0005506, GO:0016491, GO:0047938) (Fig. 5). KEGG enrichment analysis identified two significantly enriched KEGG pathways in the over expressed genes; insect hormone biosynthesis (path:00981) and ubiquinone and other terpenoid-quinone biosynthesis (path:00130) (Fig. 5).

GO and KEGG enrichment analysis were also conducted on the 202 significantly under expressed genes identifying 12 significantly enriched GO terms and one KEGG pathway (path:00981) (Fig. 5). The enriched GO terms include three terms involved in biological processes (GO:0090150, GO:0046416, GO:0006470), one involved with cellular components (GO:0005615), and seven associated with molecular functions (GO:0004866, GO:0004721, GO:0003884, GO:0032977, GO:0047938, GO:0016641, GO:0071949) (Fig. 5).

### The Overexpressed Transcriptome of Field Temephos Resistant *Aedes aegypti* Larvae

**Fig. 6.**
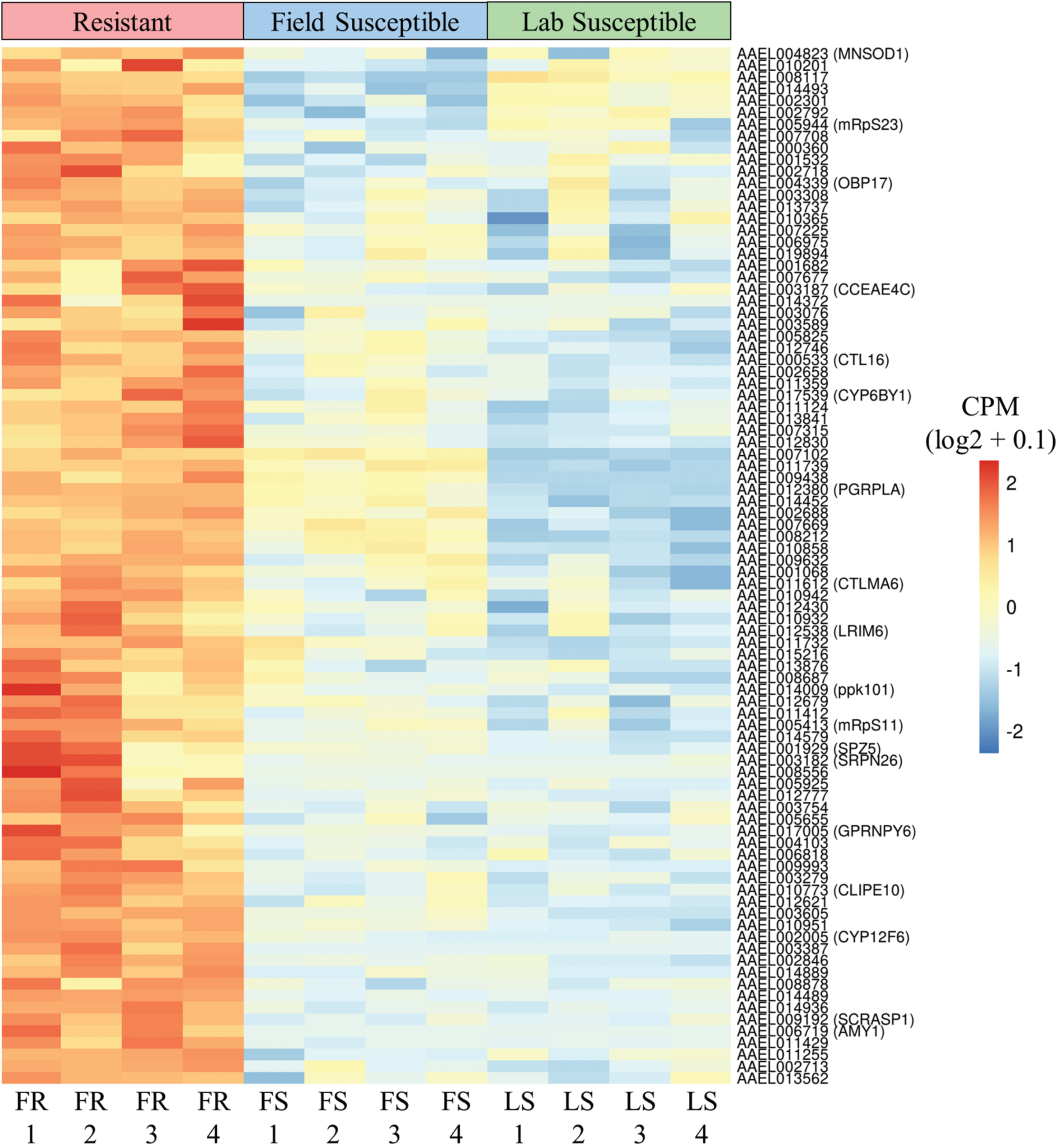
**Comparison of gene expression of significantly over expressed protein coding annotated genes in the field resistant, field susceptible, and laboratory susceptible populations.** Comparison of the FR population to both FS and LS populations identified a total of 88 protein coding VectorBase annotated genes with significant over expression (FC > 2, FDR < 0.05). The expression levels were displayed as counts per million (CPM) which is the number of counts per gene following normalization. CPM values were calculated for each gene by taking the mean CPM of each transcript within that gene. Gene expression was displayed per sample, rather than per experimental group, allowing for visualization of granularity between samples. The gene expression was scaled by row to allow comparison between samples rather than between genes. Variability in expression between samples from the same experimental groups can be seen across all genes, highlighting the importance of biological replication. There was a larger difference in expression between the FR and LS than between the FR and FS for many genes including CYP6B1 and AAEL007102, demonstrating the importance of using multiple susceptible comparator strains to reduce over estimation of DGE.

**Fig. 7.**
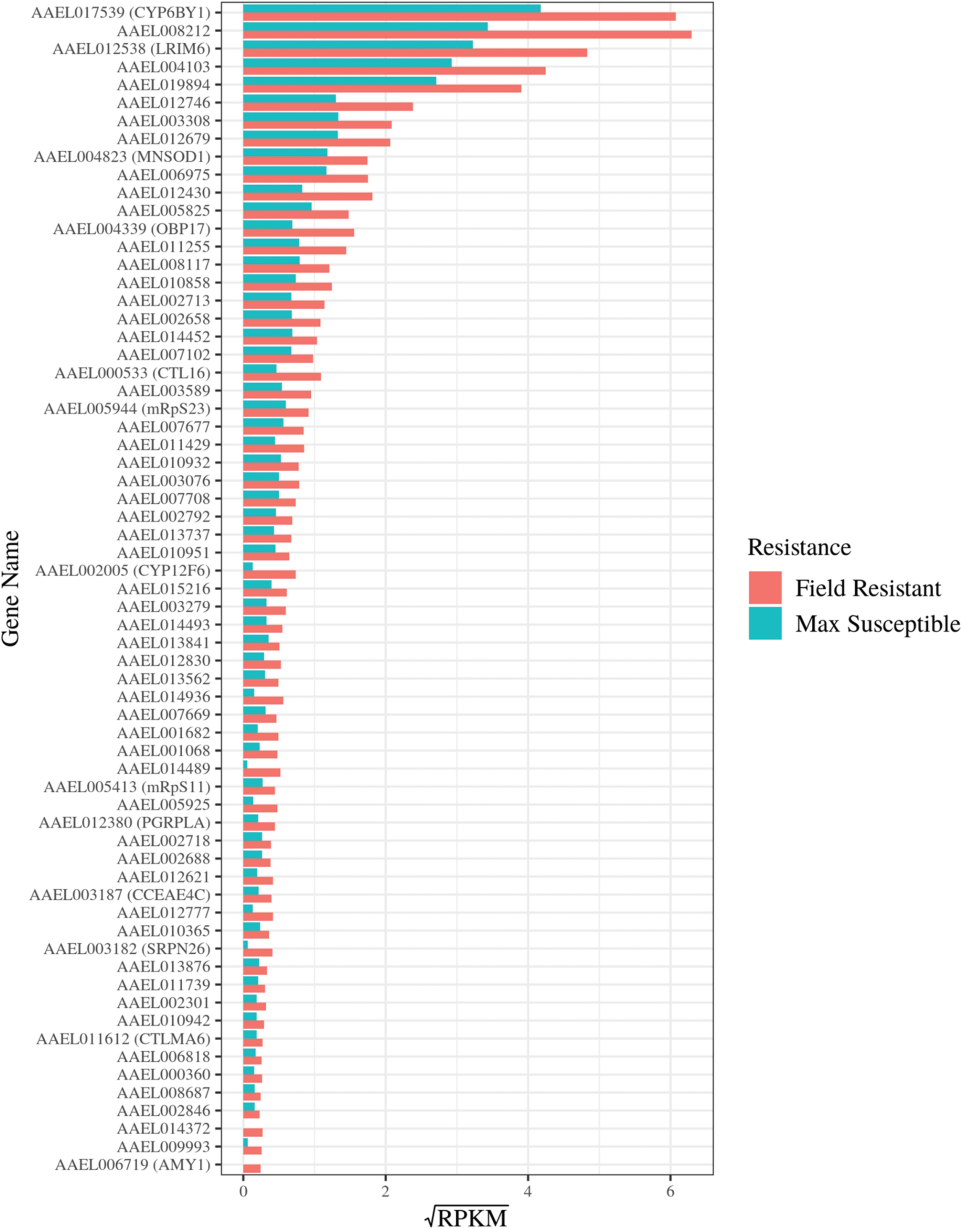
**Differences in gene expression between resistant and susceptible populations of a set of significantly over expressed, protein coding genes with VectorBase annotation.** Comparison of the FR population to both FS and LS populations identified a total of 88 protein coding VectorBase annotated genes with significant over expression (FC > 2, FDR < 0.05). Gene expression was displayed here as reads per kilobase million (RPKM) in the FR population and both susceptible populations. RPKM values were calculated for each gene by taking the mean RPKM of each transcript within that gene. The susceptible RPKMs (max susceptible) represent the maximum RPKM for each gene in both FS and LS populations. The RPKM values were square root transformed here to optimize the visualization of a vast range of values (0.02–39.11*).* Genes with average RPKM across groups of below 0.02 (23 genes) were not included in the bar plot for visualization purposes but are included in Supp Table 1 [online only]. Mean RPKM values per resistance status allow for comparison of expression between genes as well as between groups.

The transcriptomic overview provided by the GO and KEGG enrichment models was interrogated by quantifying the represented genes with CPM and RPKM metrics. The expression profiles of the 88 annotated overexpressed protein coding genes in the resistant population were visualized using heatmaps of gene expression as log_2_ values of counts per million (CPM) (Fig. 6) as well as bar plots of reads per kilobase million (RPKM) values of the represented genes (Fig. 7). The former allowed the visualization of the data’s granularity by comparing gene expression across all 16 samples individually rather than just across groups (Fig. 6). Variation in expression between samples from the same experimental group can be seen across all genes (Fig. 6) highlighting the importance of biological replication in gene expression experimentation. The heatmap also showed the importance of using field susceptible populations in addition to lab susceptible comparator populations. Differences in gene expression (e.g., *CYP6B1* and *PGRPLA*) can be overrepresented when comparing expression profiles between field and lab populations (here FR and LS) rather than between field to field (e.g., FR and FS). Differences in gene expression were also visualized using RPKM bar plots which enable ranking of genes specifically overexpressed in resistant samples, the majority (151 of the 239 protein coding genes) of those did not have a functional annotation in the current repository for VectorBase (Fig. 7, Supp Table 1 [online only]).

The over expressed annotated protein coding genes included detoxification enzymes; two cytochrome P450s (*CYP12F6 –* AAEL002005 and *CYP6BY1 –* AAEL017539), a carboxy/cholinesterase (*CCEAE4C* – AAEL003187), a glutathione S-transferase (AAEL006818), two glucosyl/glucuronosyl transferases (AAEL002688 and AAEL003076) and an aldehyde oxidase (AAEL014493). The cuticular biosynthesis enzyme chitin synthase (AAEL002718) and the digestive enzymes, putative trypsin genes, AAEL007102, AAEL014579, and AAEL003308, were also present.

Other over expressed genes included the hydrocarbon biosynthesis pathway enzyme acetyl-CoA dehydrogenase (AAEL014452) (Jones et al. 2013), glutamate decarboxylase (AAEL010951) which catalyzes biosynthesis of GABA through glutamate decarboxylation (Richardson et al. 2010), sarcosine dehydrogenase (AAEL014936), a mitochondrial glycine synthesizing enzyme (Huang et al. 2015), leucine aminopeptidase (AAEL006975), a proteolytic enzyme that hydrolyses amino acids with roles in toxin biosynthesis (Matsui et al. 2006), a manganese-iron (Mn-Fe) superoxide dismutase (*MNSOD1 –* AAEL004823), a mitochondrial antioxidant associated with increased life span in insects (Clancy et al. 2001, Kang et al. 2008) and two mannose-binding C-Type Lectins (CTLs) AAEL011612 and AAEL000533, ubiquitous proteins in multicellular organisms that provide the pattern recognition required for the initial phase of an immune response (Ourth et al. 2005, Phillips and Clark 2017).

### The Under Expressed Transcriptome of Field Resistant *Aedes aegypti* Larvae

**Fig. 8.**
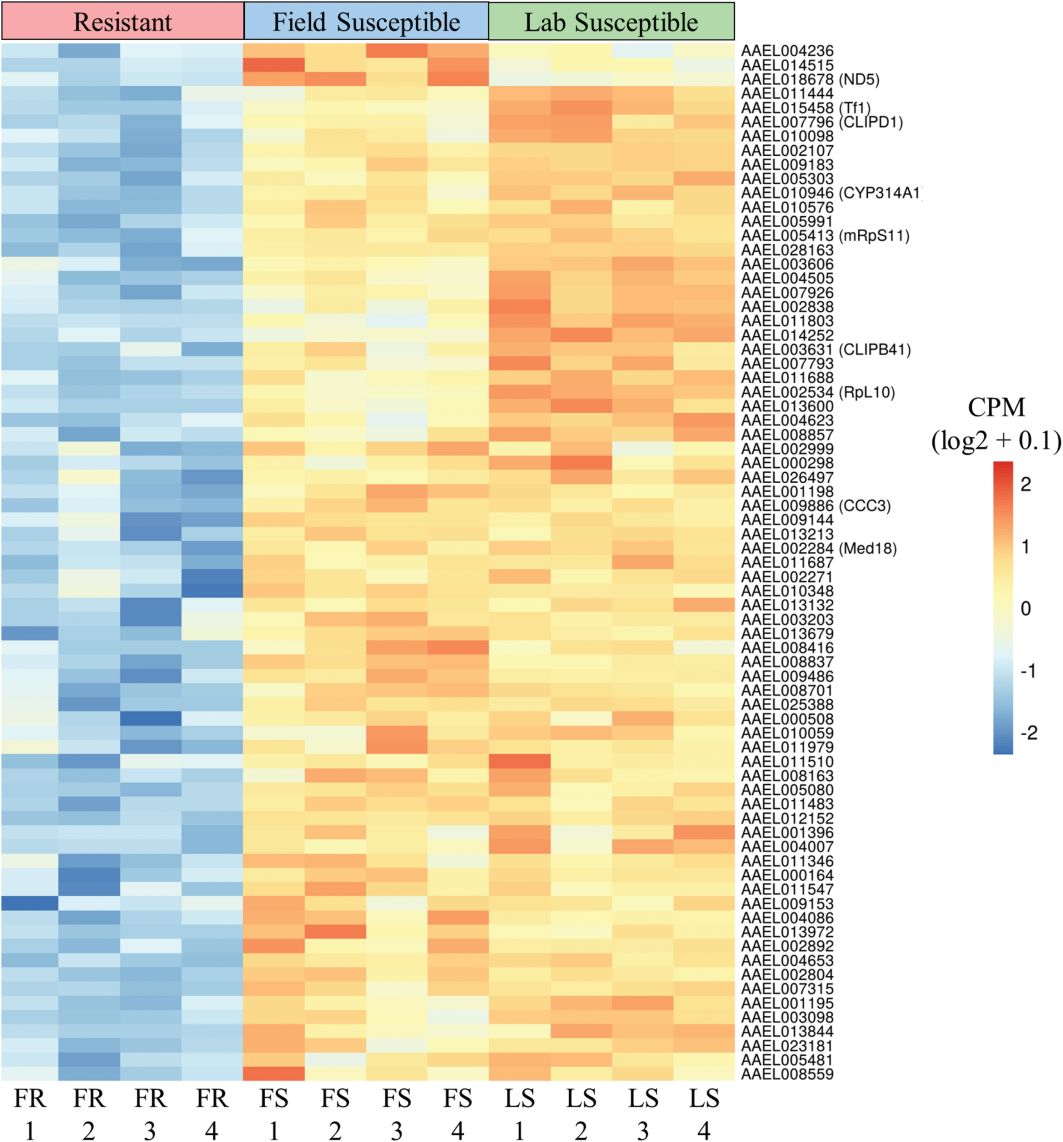
**Comparison of gene expression of significantly under expressed protein coding annotated genes in the field resistant, field susceptible, and laboratory susceptible populations.** Comparison of the FR population to both FS and LS populations identified a total of 76 protein coding VectorBase annotated genes with significant under expression (FC > 2, FDR < 0.05). The expression was displayed here as counts per million (CPM) which is the number of counts per gene following normalization. CPM values were calculated for each gene by taking the mean CPM of each transcript within that gene. Expression was displayed per sample, rather than per experimental group, allowing for visualization of granularity between samples. The expression was scaled by row to allow comparison between samples rather than between genes. Variability in expression between samples from the same experimental groups can be seen across all genes, highlighting the importance of biological replication. There was a larger difference in expression between the FR and LS than between the FR and FS for many genes including CLIPB41 and RpL10, demonstrating the importance of using multiple susceptible comparator strains to reduce over estimation of DGE.

**Fig. 9.**
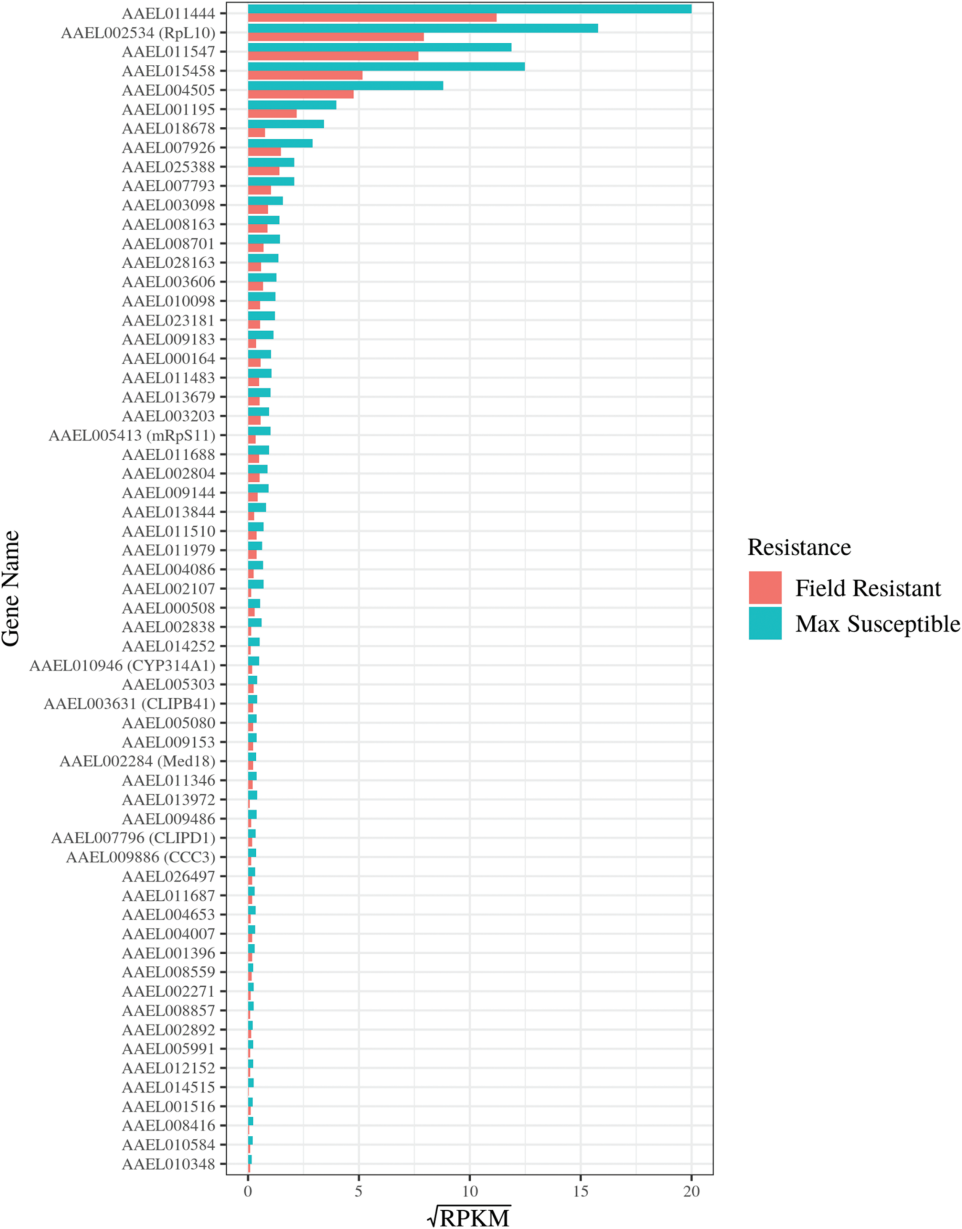
**Differences in gene expression between resistant and susceptible populations of a set of significantly under expressed, protein coding genes with VectorBase annotation**. Comparison of the FR population to both FS and LS populations identified a total of 76 protein coding VectorBase annotated genes with significant under expression (FC > 2, FDR < 0.05). Gene expression was displayed here as reads per kilobase million (RPKM) in the FR population and both susceptible populations. RPKM values were calculated for each gene by taking the mean RPKM of each transcript within that gene. The susceptible RPKMs (max susceptible) represent the maximum RPKM for each gene in both FS and LS populations. The RPKM values were square root transformed here to optimize the visualization of a vast range of values (0.02–400*).* Genes with average RPKM across groups of below 0.02 (14 genes) were not included in the bar plot for visualization purposes but are included in Supp Table 1 [online only]. Mean RPKM values per resistance status allow for comparison of expression between genes as well as between groups.

The transcript profiles of the 75 annotated protein coding genes significantly under expressed genes in the resistant population were also visualized in heatmaps of gene expression (log_2_ values of CPM) (Fig. 8) as well as bar plots of RPKMs (square root values) of the represented genes (Fig. 9). The former showing variation between the 16 samples and the latter displaying variation between genes and between resistance status.

Genes encoding detoxification enzymes were also presented in this set of under expressed genes. Those included the cytochrome P450 *CYP314A1* (AAEL010946) and a glucosyl/glucuronosyl transferase (AAEL003098). A cytochrome oxidase biogenesis protein (oxa1 mitochondrial – AAEL009183), essential for full expression of cytochrome c oxidase was also under expressed. Other genes significantly under expressed in the resistant population include a putative pupal cuticle protein (AAEL011444) and transferrin (*Tf1* – AAEL015458) a regulator of iron metabolism with roles in mosquito innate immunity (Yoshiga et al. 1997). The mdg4-binding protein ortholog gene in *Ae. aegypti* (AAEL010576: Modifier of mdg4 [Mod(mdg4)]), responsible for chromosome remodeling was also represented in this group of under expressed genes.

The expression of several ion and solute membrane transporters was also down regulated. These included the sodium-coupled cation–chloride cotransporter AAEL009886 (aeCC3), the sodium/chloride dependent amino acid transporter AAEL000298, the sodium/solute symporter AAEL001198, and the sugar transporter AAEL010348. In the group of under expressed genes were also 30 lncRNA genes in the temephos resistant larvae (Supp Table 1 [online only]).

### Gene Expression Profile of Temephos Exposed Larvae from the Resistant Population

**Fig. 10.**
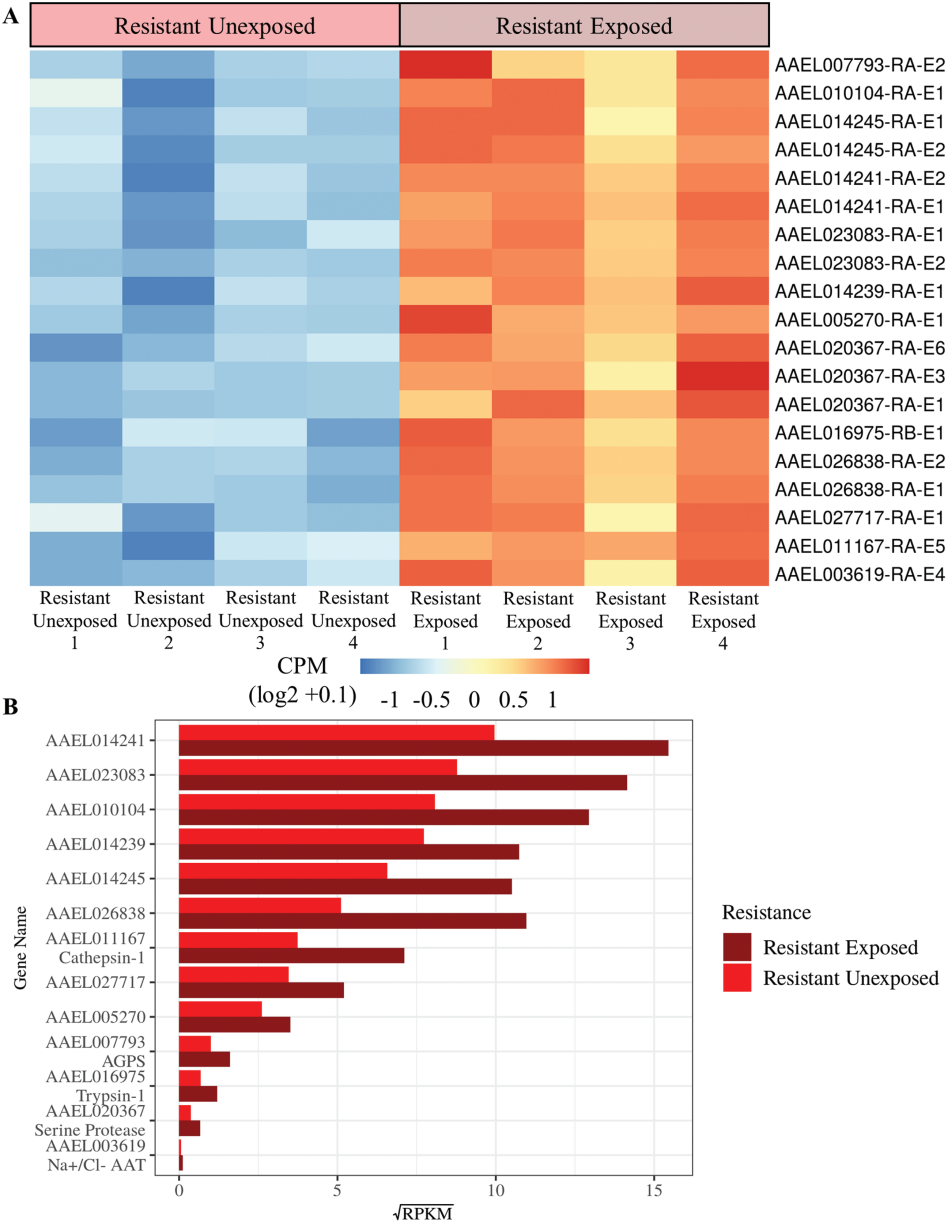
**Differential gene expression of temephos-resistant larvae under transient exposure to temephos.** Comparison of the resistant temephos exposed population with the resistant unexposed population identified 19 significantly over expressed transcripts and 13 significantly over expressed genes. The transcript expression was displayed here (A) as counts per million (CPM) which is the number of counts per transcript following normalization. Expression was displayed per sample, rather than per experimental group, allowing for visualization of granularity between samples. The expression was scaled by row to allow comparison between samples rather than between transcripts. Variability in expression between samples from the same experimental groups can be seen across all genes, highlighting the importance of biological replication. Gene expression was displayed here (B) as reads per kilobase million (RPKM) in the resistant exposed population and resistant unexposed population. RPKM values were calculated for each gene by taking the mean RPKM of each transcript within that gene. The RPKM values were square root transformed here to optimize the visualization of a vast range of values (0.003–239*).* Mean RPKM values per group allow for comparison of expression between genes as well as between groups.

Gene expression in the field resistant population following the controlled exposure to temephos was compared with gene expression of samples from the same population without insecticide exposure. The exposed samples showed 19 significantly (FC > 2, *P* < 0.05) overexpressed transcripts (Fig. 10A & Supp Table 2 [online only]) in comparison to the nonexposed samples. These 19 transcripts were mapped to 13 genes (Fig. 10). The products of the over expressed genes include a sodium/chloride dependent amino acid transporter (AAEL003619), an alkyl dihydroxyacetone phosphate synthase (AAEL007793), cathepsin-1 (AAEL011167), trypsin-1 (AAEL016975), and a serine protease stubble (AAEL020367). The remaining eight overexpressed genes had uncharacterized products in *Ae. aegypti* (Supp Table 2 [online only]).

## Discussion

Management of arbovirus burden is threatened by insecticide resistance in mosquitoes which reduces the effectivity of vector control (Maciel-de-Freitas et al. 2014, Moyes et al. 2017, Garcia et al. 2020). In this study, we report moderate resistance to temephos in the field population of *Ae. aegypti* from Cúcuta whilst larvae from Bello were susceptible. Bello is an area of relatively low arbovirus incidence (Morgan et al. 2021)and has a lower frequency of insecticide usage (Granada et al. 2018), whilst Cúcuta is an area of high arbovirus incidence which has seen routine use of temephos for *Ae. aegypti* control over four decades (Grisales et al. 2013). The reported resistance in Cúcuta is consistent with previous reports of temephos resistance in *Ae. aegypti* from Cúcuta in 2010, seven years earlier than the mosquito collections took place for this current study (Grisales et al. 2013). Whilst the resistance to temephos appears to have reduced in Cúcuta from RR_50_ = 11.85 in 2010 (Grisales et al. 2013) to RR_50_ = 8.0 in 2017 (current study) resistance to temephos remains moderate demonstrating the long-term implications of insecticide resistance on vector control programs. Management of arbovirus burden is threatened by insecticide resistance in mosquitoes which reduces the effectivity of vector control programs (Maciel-de-Freitas et al. 2014, Moyes et al. 2017), including alternatives such as biological control strategies (Garcia et al. 2020).

The triangulation of differential gene expression against two unrelated susceptible populations, one lab, and one field, was selected to reduce confounding effects of phenotypic differences between populations unrelated to insecticide resistance. Whilst this experimental design does reduce these confounding effects it is not possible to mitigate this entirely and therefore some of the differences in gene expression which are observed here may not be related to temephos resistance but resistance to other insecticides and other phenotypic differences between populations, including differences in vector competency and geographical adaptation. The differential gene expression reported here could be the reflection of the selective pressure under other larvicidal insecticides used in Cúcuta in a similar time span and even selective pressure from adulticides, such as malathion, fenitrothion, λ-cyhalothrin and deltamethrin (Ocampo et al. 2011, Maestre-Serrano et al. 2014), through vertical transfer. *Ae. aegypti* larvae from Cúcuta have previously been reported to be highly resistant to the pyrethroid λ-cyhalothrin whilst larvae from Bello were susceptible (Arévalo-Cortés et al. 2020). These findings from the same study localities used in the current study demonstrate the effect adulticide resistance can have on larvae. Cross resistance between organophosphates, such as temephos and pyrethroids has also been reported in *Ae. aegypti* (Wirth and Georghiou 1999, Rodríguez et al. 2002, Smith et al. 2019), including in Colombian *Ae. aegypti* populations (Ocampo et al. 2011).

Differential gene expression associated with the resistant phenotype was identified by selecting genes which were differentially expressed in the field resistant population compared to both the field (Bello) and lab (New Orleans) susceptible populations. This reduced the cofounding effects of location differences and enabled the analysis to focus on DGE associated specifically with resistance. This comparison identified 503 significantly differentially expressed genes which are potentially associated with the resistant phenotype, 301 of which were over expressed in the resistant population and 239 under expressed.

Genes which were found to be differentially expressed in the current study may also be the result of epistatic interactions, genetic and biochemical, and therefore associated with other biological processes aside from insecticide resistance, such as those which compensate for resistance induced fitness cost (Raymond et al. 1989, Smith et al. 2011, Hawkins et al. 2019). Epistatic interactions between genes associated with insecticide resistance are also known to influence different levels of resistance (Hardstone and Scott 2010). Under such conceptual framework the following functional categories are highlighted.

### Metabolic Detoxification Genes

Metabolic detoxification of insecticides is one of the most reported insecticide resistance mechanisms in mosquitoes. Abundant overexpression of detoxification genes, most commonly cytochrome P450 monooxygenases (P450), glutathione S-transferases (GST), and carboxylesterases (CE) has frequently been associated with insecticide resistance in mosquitoes (Vontas et al. 2020). Here we reported the overexpression of only two P450s (*CYP12F6 –* AAEL002005 and *CYP6BY1 –* AAEL017539), one GST (AAEL006818) and one CE (*CCEAE4C* [AAEL003187]), in the resistant population compared to both field and lab susceptible populations. CYP12F6 has previously been shown to be overexpressed in a permethrin resistant population of adult *Ae. aegypti* from Mexico albeit compared with a lab susceptible population only (David et al. 2014). GO terms associated with insecticide detoxification (oxidoreductase activity (GO:0016491) and oxidation–reduction process [GO:0055114]) were also found to be enriched in the temephos resistant larvae. Thus, by cross examining the data in field-to-field and field-to-lab population comparison, we observed genes representing these three forms of insecticide deactivation in much reduced number compared to what is commonly reported (Marcombe et al. 2009, Grisales et al. 2013, Dusfour et al. 2015, Ishak et al. 2017). To illustrate the above, if the resistant population had been compared with the lab susceptible population only a total of 49 cytochrome P450s, six GTSs, and 11 CEs would have been reported as differentially expressed (Supp Table 3 [online only]). This suggests that large overexpression of detoxification genes may be partly related to differences between field and lab mosquitoes rather than associated with the insecticide resistant phenotype. Large overexpression of detoxification in mosquitoes may also only be observed in mosquitoes when they have high levels of resistance rather than the moderate resistance reported here (Dusfour et al. 2015, Ishak et al. 2015).

### Chitin Biosynthesis

The thickness and composition of the cuticle have been identified as a critical determinant of insecticide resistance due to its role in reducing insecticide penetration (Balabanidou et al. 2018). Over expression of genes associated with formation and maintenance of the cuticle have been reported in insecticide resistant populations of medically relevant species including *An. gambiae* (Awolola et al. 2009, Balabanidou et al. 2016, Yahouédo et al. 2017), *An. funestus* (Giles, Diptera: Culicidae) (Gregory et al. 2011) and *Culex pipiens pallens* (Linnaeus, Diptera: Culicidae) (Pan et al. 2009, Fang et al. 2015). The cuticle has also been associated with resistance in *Ae. aegypti* including in larvae (David et al. 2010, Riaz et al. 2013). The over expression of the chitin biosynthesis enzyme AAEL002718 and the enrichment of chitin synthase activity (GO:0004100) in temephos resistant *Ae. aegypti* larvae reported in this study further highlights the potential role of the cuticle in the development of insecticide resistance in *Ae. aegypti* larvae, even when resistance is moderate. Chitin, a biopolymer of N-acetylglucosamine, is a major constituent of the mosquito cuticle (exoskeleton [epidermal cuticle], tracheal cuticle, and eggshell) providing it with both strength and rigidity and is also found in midgut peritrophic matrices (Merzendorfer and Zimoch 2003). The use of chitin synthesis inhibitors (CSI), a type of insect growth regulators (IGRs) which interfere with the synthesis and deposition of chitin on the exoskeleton (Tunaz and Uygun 2004), has been highlighted as a potential approach to control *Ae. aegypti* with some promising findings in laboratory studies (Martins et al. 2008, Fontoura et al. 2012).

### Pattern Recognition and Innate Immunity

Temephos resistant *Ae. aegypti* larvae were shown to express high levels of the mannose-binding C-type lectins (CTLs) AAEL011612 and AAEL000533 which are predominantly produced in the salivary glands of adult female *Ae. aegypti* (Ribeiro et al. 2016, Adelman and Myles 2018). Lectins are ubiquitous proteins in multicellular organisms that provide the pattern recognition required for the initial phase of an immune response (Ourth et al. 2005, Phillips and Clark 2017). C-type lectins are a group of calcium-dependant carbohydrate binding proteins (Zelensky and Gready 2005). In mosquitoes CTLs are primarily involved in facilitating viral infection (e.g., dengue, Rift Valley fever and Japanese encephalitis viruses (Liu et al. 2017, Licciardi et al. 2020)) through the enhanced viral entry, acting as bridges between flaviviruses and host cell receptors (Cheng et al. 2010, Liu et al. 2017). However, these proficient pattern recognition proteins seem to have evolved to mediate multiple multicellular processes beyond mosquito immune response including lifespan and reproductive capability (Li et al. 2020) as well as maintenance of gut microbiome homeostasis (Pang et al. 2016). Transferrin (*Tf1* – AAEL015458), found to be under expressed in temephos resistant larvae in the current study, also has roles in the innate immune response to arbovirus infection (Wessling-Resnick 2018, Licciardi et al. 2020) and has previously been reported to be down regulated in CHIKV and DENV infected mosquitoes which may favor viral replication (Tchankouo-Nguetcheu et al. 2010). Transferrin expression has also been related to insecticide resistance in *Culex pipiens* with increased expression reported in mosquitoes with target-site resistance to pyrethroids and organophosphates, the biggest difference in transferrin expression was observed in adults (Tan et al. 2012, Vézilier et al. 2013).

### Cell Membrane Transport

The expression of several ions coupled solute membrane transporters was down regulated in temephos resistant larvae: the sodium-coupled cation–chloride cotransporter AAEL009886 (aeCC3), the sodium/chloride dependent amino acid transporter AAEL000298, the sodium/solute symporter AAEL001198, and the sugar transporter AAEL010348. aeCCC3 is a larvae specific membrane transporter abundant in the anal papillae responsible for the absorption of external ions (Piermarini et al. 2017) which belongs to a family of cation-coupled chloride cotransporters (CCCs) which contribute to ion homeostasis by undertaking electroneutral transport of Na^+^, K+, and Cl^−^ (Pullikuth et al. 2003). A similar role is expected from the ion-coupled transporters AAEL000298 and AAEL001198 in the homeostasis of ion content, particularly in the midgut and Malpighian tubes where they are most abundant (Li et al. 2017).

The aquatic life of the *Ae. aegypti* larval stages demands an ion exchange homeostasis that differs from that of the adult mosquitoes. Due to their freshwater habitat *Ae. aegypti* larvae must excrete water gained by osmosis, reabsorb salt before excreting urine, and absorb salt from their surroundings (Ramasamy et al. 2021). Whilst the opposite is true in adults where water retention is needed due to constant loss through evaporation. A key process in this is Na+-dependent co-transport which is typically down the large inward (extracellular to intracellular) Na+ gradient generated by the Na+/K+-ATPase (Payne 2012). We speculate that the ion homeostasis changes caused by the reduced expression of the three CCCs transporters AAEL009886, AAEL000298, and AAEL001198 could reduce the exposure of larvae to temephos by reducing net uptake of the molecule, protecting the organs where they are commonly expressed (e.g., midgut and Malpighian tubes). Transcriptome studies of insecticide resistant mosquito populations tend to overlook the potential role of down regulated genes in favor of overexpressed genes, but this finding demonstrates the importance of investigating reduced expression when studying potential mechanisms of insecticide resistance. The potential role of CCC transporters in reducing insecticide uptake and therefore facilitating resistance warrants further investigation.

### Chromosomal Remodeling

The mdg4-binding protein ortholog gene (AAEL010576: modifier of mdg4 [Mod(mdg4)]), responsible for chromosome remodeling was also significantly under expressed in the resistant *Ae. aegypti* larvae. Originally described as a protein binding the transposon mdg4 (Bayev et al. 1984), Mod(mdg4) gene encodes for a family of proteins due to at least 30 different alternative splicing variants in Diptera and Lepidoptera (Krauss and Dorn 2004, Gabler et al. 2005, Tikhonov et al. 2018). Mod(mdg4) variants bind a variety of insulators (DNA domains involved in nuclear organization and higher order chromatin structures) (Ghosh et al. 2001, Melnikova et al. 2004, Golovnin et al. 2007) and have been involved in regulating numerous traits of the insect embryonic progression such as synapsis structure (Gorczyca et al. 1999), chromosome Y-linked testis development (Branco et al. 2013), and midgut maturation (Cai et al. 2012). Changes in expression of Mod(mdg4) have been reported in *Drosophila* Kc cells treated with deltamethrin (Liu et al. 2020). The downstream targets of mod (mdg4) have also been shown to affect the down regulation of proteins associated with protection from various stress conditions, therefore acting as modulators of cytotoxic damage in *Drosophila* (Lin et al. 2021). The identification of under expression of the mdg4-binding protein in temephos resistant larvae suggests a further role for this protein in mediating insecticide resistance with a potential role in regulating the stress response.

### Long Noncoding RNA

There were 55 over expressed and 30 under expressed genes encoding for long noncoding RNA (lncRNA) in the temephos resistant larvae (Supp Table 1 [online only]). Noncoding RNAs (ncRNA) are abundant cellular effectors of great prolific functionality (Eddy 2001) and long ncRNA are defined as transcripts, more than 200 nucleotides long, that are produced by RNA polymerase II and are not translated into proteins (Bonasio and Shiekhattar 2014). In *Aedes* lncRNAs, mainly involved in regulating gene expression, are multifunction with roles including sex differentiation (Xu et al. 2019), embryogenesis (Azlan et al. 2019), and suppression of viral replication in DENV infected mosquitoes (Etebari et al. 2016). Long ncRNAs have also been associated with insect’s response to xenobiotics, with reports of differential lncRNA expression in resistant populations of *Plutella xylostella* (Etebari et al. 2015). The findings of 85 differentially expressed lncRNAs reported here in resistant populations of *Ae. aegypti* supports the potential roles that lncRNAs could have in the development of insecticide resistance. Whilst gene expression studies have focused primarily on differential expression in protein coding genes, the development of next generation techniques has now provided an opportunity to also study noncoding RNA. Whilst work has been conducted into identifying lncRNAs in medically relevant mosquito species including *An. gambaie* (Jenkins et al. 2015) and *Ae. aegypti* (Azlan et al. 2019) there have been no studies that have aimed to investigate the role of lncRNAs in insecticide resistance in mosquitoes. Previous RNA-Seq studies on insecticide resistant populations of *Culex pipiens pallens* have also identified differential expression of lncRNAs (Shi et al. 2020), however, an in-depth discussion of their role in insecticide resistance has been neglected.

### Differential Gene Expression in Resistant Larvae Following Temephos Exposure

In the study, we also tracked gene expression in insecticide resistant larvae following direct response to temephos exposure. Thirteen genes were found to have a significantly increased expression following a controlled exposure to temephos. Among those 13 genes were two serine proteases: trypsin −1 (AAEL016975) and serine protease stubble (AAEL020367), a cysteine protease: cathepsin-1 (AAEL011167), a sodium/chloride dependent amino acid transporter (AAEL003619), and an alkyl dihydroxyacetone phosphate synthase (AAEL007793). Serine proteases are a group of enzymes with a variety of known functions including digestion, metamorphosis, oogenesis, blood coagulation, and viral immune response (Terra and Ferreira 1994, Mesquita-Rodrigues et al. 2011, Licciardi et al. 2020). Cathepsin-1 (AAEL011167), a cysteine proteinase, is also a multifunctional digestive enzyme (Terra and Ferreira 1994, Liu et al. 2006). Upregulation of serine proteases has been previously reported in insecticide resistant mosquito populations (Bonizzoni et al. 2012, Reid et al. 2012, David et al. 2014, Zou et al. 2016), including temephos resistant *Ae. aegypti* from Cúcuta (Grisales et al. 2013). Serine proteases have also been shown to degrade insecticides through hydrolysis within the insect digestive tract, however, so far evidence of this is limited to pyrethroids such as deltamethrin (Yang et al. 2008a, b; Xiong et al. 2014; Wilkins 2017). Overexpressed proteases in this current study support the findings of previous studies that proteases may have a role in the metabolism of other insecticide classes besides pyrethroids. The changes responsible for resistance are often associated with modification of physiological processes that can lead to decreased performance and fitness disadvantage.

Deleterious effects of insecticide resistance can affect a wide range of life-history traits (e.g., longevity, biting behavior, and vector competence) (Alout et al. 2014, 2017). Although the cost of resistance genes is believed to gradually decrease due to subsequent modifier mutations (Raymond et al. 2001). With the relatively limited diversity of insecticide targets (Swale 2019), the gene expression patterns that resistant mosquitoes further undergo when exposed to the insecticide could be a source for novel assets for vector control. The study of such targets for insecticide development is a strategy that, to our knowledge, has not yet been explored.

## Conclusion

This study found differential insecticide responses from *Ae. aegypti* field samples of two previously epidemiologically characterized sites in Colombia. Using these contrasting *Ae. aegypti* field mosquito populations together with the New Orleans lab strain, we demonstrated the risk of producing noise signal by overestimating by several fold the differential gene expression if mosquito populations are compared only with laboratory strains. The two overexpressed P450s in resistant *Ae. aegypti* larvae represent some ten-fold lower levels in comparison to previous studies (Dusfour et al. 2015, Ishak et al. 2015). The results also provide an insight into expression changes that are observed in moderately resistant mosquitoes as a useful insight into the biology of the progression of resistance. The role of the cuticle in insecticide resistance suggested in previous studies is substantiated here. This study also identified several genes potentially associated with the resistant phenotype that have not been previously associated with insecticide resistance in mosquitoes. These included changes in ion exchange homeostasis, chromatin remodeling, lectin-mediated immune responses, and a plethora of lncRNAs. Evidently, there is a notorious gap in our knowledge base of gene expression adaption in insecticide resistance. The work presented here contributed to what seems to be an expansive and varied phenotypic landscape in the *Ae. aegypti* responses to insecticides of current importance.

## Supplementary Data

Supplementary data are available at *Journal of Medical Entomology* online.

Supp Table 1. **Significantly differentially expressed genes and transcripts in FR samples when compared to FS and LS samples.** A total of 379 transcripts covering 301 genes were significantly over expressed and 244 transcripts covering 202 genes were significantly under expressed in the resistant population when compared to both susceptible populations. Genomic location, product description, gene type, and gene name/symbol obtained from VectorBase annotations. Reads per kilobase million (RPKM) for each population, fold change (logFC), counts per million (logCPM), F-test statistic (F), *P* value, and false discovery rate (FDR) calculated using edgeR.

Supp Table 2. **Significantly differentially expressed genes and transcripts in the resistant population following temephos exposure.** A total of 19 transcripts covering 13 genes were significantly over expressed in the temephos exposed resistant population when compared to unexposed resistant larvae. Genomic location, product description, gene type and gene name/symbol obtained from VectorBase annotations. Reads per kilobase million (RPKM) for each population, fold change (logFC), counts per million (logCPM), F-test statistic (F), *P* value, and false discovery rate (FDR) calculated using edgeR. Uncharacterized genes were searched for homologs in other species using NCBI nucleotide blast (https://blast.ncbi.nlm.nih.gov/Blast.cgi), but no characterized homologs were identified.

Supp Table 3. **Significantly differentially expressed genes and transcripts in FR samples when compared to FS samples only.** A total of 3328 transcripts covering 2322 genes were significantly over expressed and 2250 transcripts covering 1555 genes were significantly under expressed in the resistant population when compared to the lab susceptible population. Genomic location, product description, gene type, and gene name/symbol obtained from VectorBase annotations. Reads per kilobase million (RPKM) for each population, fold change (logFC), counts per million (logCPM), F-test statistic (F), *P* value, and false discovery rate (FDR) calculated using edgeR.

tjab179_suppl_Supplementary_Table_S1

tjab179_suppl_Supplementary_Table_S2

tjab179_suppl_Supplementary_Table_S3
